# *In silico* approaches in carcinogenicity hazard assessment: case study of pregabalin, a nongenotoxic mouse carcinogen

**DOI:** 10.3389/ftox.2023.1234498

**Published:** 2023-11-13

**Authors:** Douglas A. Keller, Arianna Bassan, Alexander Amberg, Leigh Ann Burns Naas, Jon Chambers, Kevin Cross, Frances Hall, Gloria D. Jahnke, Amarjit Luniwal, Serena Manganelli, Jordi Mestres, Amy L. Mihalchik-Burhans, David Woolley, Raymond R. Tice

**Affiliations:** ^1^ Independent Consultant, Kennett Square, PA, United States; ^2^ Innovatune, Padova, Italy; ^3^ Sanofi Preclinical Safety, Frankfurt, Germany; ^4^ Magnolia Toxicology Consulting, Traverse City, MI, United States; ^5^ Instem, Cambridge, United Kingdom; ^6^ Instem, Columbus, OH, United States; ^7^ Independent Consultant, Chapel Hill, NC, United States; ^8^ Medline Industries, Northfield, IL, United States; ^9^ Nestlé Research, Lausanne, Switzerland; ^10^ Chemotargets SL, Parc Científic de Barcelona, Barcelona, Spain; ^11^ ToxStrategies LLC, Asheville, NC, United States; ^12^ ForthTox, Linlithgow, United Kingdom; ^13^ RTice Consulting, Hillsborough, NC, United States

**Keywords:** In silico toxicology protocol, mode of action, pregabalin, non-genotoxic carcinogen, oxidative stress, chronic inflammation, cell proliferation

## Abstract

*In silico* toxicology protocols are meant to support computationally-based assessments using principles that ensure that results can be generated, recorded, communicated, archived, and then evaluated in a uniform, consistent, and reproducible manner. We investigated the availability of *in silico* models to predict the carcinogenic potential of pregabalin using the ten key characteristics of carcinogens as a framework for organizing mechanistic studies. Pregabalin is a single-species carcinogen producing only one type of tumor, hemangiosarcomas in mice via a nongenotoxic mechanism. The overall goal of this exercise is to test the ability of *in silico* models to predict nongenotoxic carcinogenicity with pregabalin as a case study. The established mode of action (MOA) of pregabalin is triggered by tissue hypoxia, leading to oxidative stress (KC5), chronic inflammation (KC6), and increased cell proliferation (KC10) of endothelial cells. Of these KCs, *in silico* models are available only for selected endpoints in KC5, limiting the usefulness of computational tools in prediction of pregabalin carcinogenicity. KC1 (electrophilicity), KC2 (genotoxicity), and KC8 (receptor-mediated effects), for which predictive *in silico* models exist, do not play a role in this mode of action. Confidence in the overall assessments is considered to be medium to high for KCs 1, 2, 5, 6, 7 (immune system effects), 8, and 10 (cell proliferation), largely due to the high-quality experimental data. In order to move away from dependence on animal data, development of reliable *in silico* models for prediction of oxidative stress, chronic inflammation, immunosuppression, and cell proliferation will be critical for the ability to predict nongenotoxic compound carcinogenicity.

## 1 Introduction

Cancer is a multifaceted, multimodal disease. Whereas advances in cancer treatment over the last five decades have been remarkable (Arnold, et al., 2019; Kratzer, et al., 2022), many causes of cancer in the human population are still largely unknown. Given that there are tens of thousands of chemicals in commerce that have not had adequate carcinogenicity testing, there is a need for a swift and reliable assessment of the carcinogenic potential of chemicals. Over 50 years after coming into common use, the 2-year rodent carcinogenicity bioassay is still considered by many regulatory authorities, legislative bodies, industrial entities and other authoritative groups to be the gold standard for assessment of carcinogenicity. This bioassay has many flaws, including low sensitivity, dose levels that are often irrelevant to human exposure, expense, and high animal usage (Cohen, 2017; Goodman, 2018; Madia, et al., 2019). The environmental and agrochemical sectors generally require 2-year rat and mouse studies for carcinogenicity assessment of new chemical entities, with varying levels of acceptance of mechanistic data to modify risk assessment across regulatory bodies. The 6-month transgenic mouse assay has largely supplanted the 2-year mouse study in pharmaceutical development, and the International Council for Harmonisation of Technical Requirements for Pharmaceuticals for Human Use (ICH) S1B(R1) guideline allows for a weight of evidence (WoE) assessment to determine the need for a 2-year rat study. However, replacement of the bioassay with alternative methods including *in vitro* or computational tools has not been well accepted as a definitive tool for risk assessment and regulatory purposes.

There are many theories about the origins of cancer and multiple attempts have been made to categorize chemicals into classes of carcinogens for the purposes of hazard or risk assessment (Doll and Peto, 1982; Wolf et al., 2019). Classification systems such as those from the International Agency for Research on Cancer (IARC) and the US National Toxicology Program (NTP) are generally hazard classification systems, with little account for exposure to assess risk. Other classification systems focus mode of action (MOA) (Boobis et al., 2006; Cohen et al., 2019) and also account for exposure where key characteristics are altered to give an estimate of risk. Smith et al. (2016, Smith et al., 2020) describe the ten key characteristics (KC) of carcinogens as an approach to evaluate mechanistic evidence in cancer hazard identification. Many of the KCs described by Smith et al. are also part of the assessment system proposed by Cohen et al. (2019) but are described in more detail and with proposed experimental methods of assessment in the Smith papers. The KCs are a method that can be used to organize data relevant to the MOA of a carcinogen, and to provide a systematic evaluation of cancer hazards.

Tice, et al. (2021) extended these concepts and described the current status and future needs for *in silico* carcinogenicity assessment based on the attributes of the KCs of carcinogens (Smith, et al., 2016; Smith, et al., 2020). In this context, *in silico* (computational) approaches refer to different methodologies that aim at predicting adverse effects from the structure of molecules. These approaches are based on structure activity relationships (SARs) between structural information and biological activity; SARs may be either qualitative or quantitative in nature and are commonly referred to as (Q)SARs. Tice, et al. (2021) make clear that additional *in silico* models are needed to describe many of the KCs for carcinogens in order to expedite the analysis of the potential carcinogenicity of the many thousands of chemicals in commerce (Tice, et al., 2021). Moreover, the ultimate goal is the integration of such *in silico* approaches in a hazard assessment framework of carcinogens in a transparent, consistent, and defendable manner.

This view follows the *in silico* toxicology (IST) protocol initiative for the development of standardized approaches for the prediction of toxicity from a chemical structure (Myatt, et al., 2018; Myatt, et al., 2022). Similar to the published test guidelines for *in vivo* or *in vitro* test methods, the IST protocols are meant to support *in silico* assessments using principles that ensure that results can be generated, recorded, communicated, archived, and then evaluated in a uniform, consistent, and reproducible manner. The protocols define the effects and/or mechanisms to be predicted by the *in silico* methods as part of the assessment of interest. They describe how the data are combined to assess one or more endpoints including creation of an overall confidence score based on a weight of evidence. To further illustrate the needs for *in silico* model development in support of carcinogenicity assessment as well as to gain knowledge for the development of a corresponding IST protocol, an international consortium has undertaken a series of case studies of chemicals and drugs with varying carcinogenicity bioassay outcomes. This consortium workgroup builds upon the efforts that were undertaken to evaluate the extent to which *in silico* models exist for each of the 10 KCs (Tice, et al., 2021).

Here, we report on pregabalin, a compound that is carcinogenic in mice via a nongenotoxic mechanism (Pegg, et al., 2012). The overall goal of this exercise is to test the ability of *in silico* models to predict nongenotoxic carcinogenicity with pregabalin as a case study. Available experimental data and computational model predictions are organized in terms of the KC framework, and gaps in the availability of models are discussed. The 10 KCs conceptual framework (Smith, et al., 2016; Smith, et al., 2020) offers a construct that supports the expert review of available evidence, with a focus on the ability of *in silico* tools to predict the outcome of carcinogenicity studies. Smith, et al. (2020) also present a summary of *in vitro* assays and *in vivo* biomarkers that can be used to investigate certain aspects of modes of action (MOAs) and the KCs.

Insights from Adverse Outcome Pathways are also integrated in such a process. Nongenotoxic rodent carcinogenicity is often considered to be non-relevant to human health (Silva Lima and van der Laan, 2000), but significant experimental research is needed to substantiate that claim. The use of *in vitro* and *in silico* tools to support this process could increase the speed of research and decrease animal use.

The following sections describe, for each of the ten KCs of carcinogens, a summary of the available data for pregabalin, computational modeling of the KC, and modeling and data gaps. Additional details of these data can be found in the references cited. Since pregabalin was not carcinogenic in rats at up to 14 (males) and 24 (females) times the maximum recommended human dose, data described in the KC focus on data from studies in mice or *in vitro*. Confidence in these data and computational approaches is assigned based on criteria described by Johnson et al. (Johnson, et al., 2022).

## 2 Materials and methods

### 2.1 Chemical information

**FIGURE 1:**
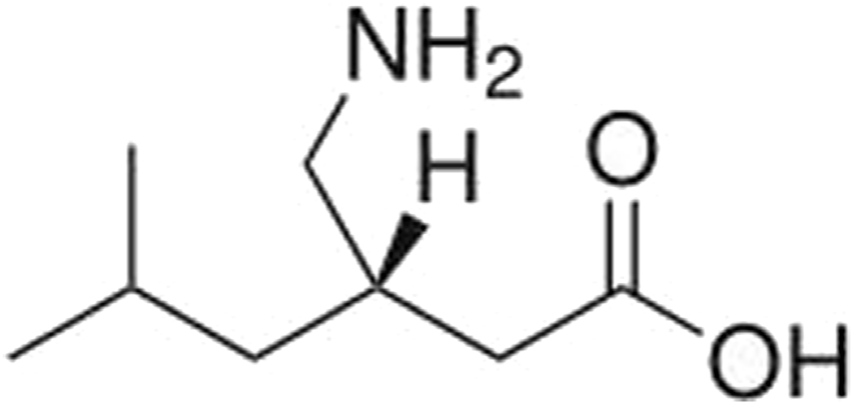
Chemical structure of pregabalin.

Pregabalin (CAS Number 148553-50-8; Figure 1) is a small molecule ligand of the auxiliary α2δ subunit site of certain voltage-dependent calcium channels (VDCCs), and acts as an inhibitor of α2δ subunit-containing VDCCs. It is used to treat seizures, neuropathic pain, and generalized anxiety disorders (Mico and Prieto, 2012; Alles, et al., 2020) and is globally available. The molecular weight of pregabalin is 159 g/mol and the log P is 1.3. It is freely soluble (high solubility) in water.

### 2.2 Metabolism data

Pregabalin is rapidly absorbed orally, with a bioavailability of approximately 80%. There is no significant metabolism in humans or other species, with the exception of the dog. The major *in vivo* metabolite is N-methyl pregabalin, accounting for <3% of drug-related material in most species, and approximately 45% of drug-related material in the dog. The principal route of excretion is in the urine. Pregabalin is not a CYP inhibitor *in vitro* at concentrations up to 1 mM (EMA, 2005).

### 2.3 Carcinogenicity data

During development, pregabalin was tested for potential carcinogenicity in 2-year bioassays in mice and rats (Pegg, et al., 2012). Pregabalin did not induce tumors in rats but did in mice. The induced tumors in mice were hemangiosarcomas, primarily in the spleen, liver, and bone marrow. Additional studies were performed to elucidate the mechanism of hemangiosarcoma formation and potential human relevance (Criswell, et al., 2012a; Criswell, et al., 2012b; Criswell, et al., 2012c). These studies showed that pregabalin induces hemangiosarcomas through increased hypoxia and endothelial cell (EC) proliferation in a species-specific manner. In addition to these studies on mice, rats were included in many studies to validate that the effects observed and the mode of action were specific to the mouse.

A search for potency (ChEMBL, 2022) values for pregabalin and structurally similar compounds was performed in ChEMBL (release 31). The Comparative Toxicogenomics Database (CTDB, 2022) was searched for data on pregabalin potentially related to mechanisms of carcinogenicity. The ClarityPV platform (CLARITY, 2023) was searched for neoplasm side effects associated with pregabalin use.

*In silico* predictions for pregabalin carcinogenicity and carcinogenicity potency were obtained from commercially and freely available *in silico* platforms (Leadscope Model Applier (v. 3.1.0-40), Derek Nexus 6.1.0, VEGA v. 1.3.10, Toxtree v.3.1.0, LAZAR v. 1.4.2).

### 2.4 Key characteristics

The available experimental data and *in silico* predictions for pregabalin were reviewed by organizing such information within the KC of carcinogens framework. Data were assessed in terms of the reliability score (RS) and relevance as discussed by Myatt et al. (Myatt, et al., 2018) and Johnson et al. (Johnson, et al., 2022), that, at the experimental level, may consider different factors such as compliance with guidelines, concordance with other studies, and/or deviations from test protocols (see Table 1). At the *in silico* prediction level, reliability refers to the extent that an *in silico* result is predictive of an experimental result. On the other hand, the expert conclusions that integrate and combine evidence from various experimental or *in silico* results (each of this can be associated with a specific RS) can be scored according to the confidence categories (high, medium, low, or no confidence), that have been specifically developed for a given toxicological assessment by Johnson et al. (Johnson, et al., 2022). Table 1 summarizes the scoring system adopted in the current work for assessing the reliability of available experimental data and *in silico* predictions (reliability scores) and for assessing the confidence of the conclusions related to the key characteristics (confidence categories).

**TABLE 1:** Reliability scores (RSs) and confidence categories used to respectively assess data and conclusions on KCs in the present work. The RS is applied for assessing experimental data and *in silico* predictions (Myatt, et al., 2018; Johnson, et al., 2022); the RS framework integrates the Klimish scoring system for experimental data (Klimisch, et al., 1997). The confidence categories have been developed to grade the confidence of the assessment of a toxicological endpoint (Johnson, et al., 2022) and they can be applied here to grade the expert conclusions related to the key characteristics of carcinogens.

Reliability of experimental data and *in silico* predictions	
Reliability score	Definition
RS1	Experimental data that are well documented and accepted; data from study performed according to valid and/or accepted test guidelines, preferably following good laboratory practices (GLP). RS1 is not assigned to *in silico* predictions
RS2	Experimental data that are well documented and sufficient; data generally from study not following GLP; partially compliant with test guideline. RS2 is not assigned to *in silico* predictions
RS3	Expert review of available evidence as coming from *in silico* predictions (including read-across) and/or from low reliability experimental studies
RS4	Multiple *in silico* predictions that are in agreement
RS5	Single acceptable *in silico* result or Experimental data not reliable

Confidence of the assessment as coming from combining different pieces of evidence	
Confidence category	Definition
High	A high confidence of the assessment suggests that sufficient evidence is available to support an accurate conclusion, and further research is unlikely to increase the confidence
Medium	A medium confidence of the assessment suggests adequate evidence is available to support an accurate conclusion, but further research might increase the confidence
Low	A low confidence of the assessment suggests that available evidence is lacking to support an accurate conclusion and further research is required to derive any robust conclusion and to improve its confidence
No confidence	A no confidence of the assessment suggests that further research is required for the derivation of an assessment

## 3 Results

### 3.1 Key characteristics

#### 3.1.1 KC1: Is electrophilic or can be metabolically activated

##### 3.1.1.1 Experimental data

Pregabalin is not electrophilic based on its absence of activity in bacterial mutagenicity assays that incorporate metabolic activation, which are considered to be an acceptable surrogate for the electrophilicity endpoint (Ashby and Tennant, 1988). Furthermore, the lack of significant metabolism in all species tested except for the dog supports a lack of concern about a possible electrophilic metabolite, as none could be generated.

##### 3.1.1.2 *In silico* approaches

Only one metabolite of pregabalin, N-methylpregabalin, has been experimentally isolated and it is a minor component, comprising only a few percent of the dose in all species except the dog (Pharmapendium, 2022). *In silico* predictions for bacterial mutagenicity of N-methyl pregabalin suggested the absence of electrophilicity character. Details of the *in silico* predictions are reported in the Supplementary Material.

##### 3.1.1.3 Reliability and confidence

The experimental data are assigned a reliability score of RS1. The *in silico* prediction for N-methyl pregabalin is assigned a reliability score of RS3. Given the reliability of available evidence and its corresponding relevance for the evaluation of electrophilicity, a medium confidence can be assigned to the conclusion that pregabalin is not electrophilic (or cannot be metabolized to active intermediates).

##### 3.1.1.4 Data gaps

Standard regulatory metabolism studies were performed with pregabalin and are complete for animal species. Additional human metabolism data would increase the confidence in extrapolation of the animal data to humans and better clarify the human relevance. Specific *in vitro* experiments for electrophilicity endpoints would be useful.

#### 3.1.2 KC2: Is genotoxic

##### 3.1.2.1 Experimental data

Several genotoxicity studies using standard Organisation for Economic Co-operation and Development (OECD) test guideline assays were submitted to the U.S, Food and Drug Administration (FDA) during the registration of pregabalin (Pegg, et al., 2012). In bacterial reverse mutation assays, pregabalin was reported negative in *Salmonella typhimurium* strains TA-98, TA-100, TA-1535, and TA-1537 at maximal concentrations of 5,000 µg/plate, with and without metabolic activation, and in *Escherichia coli* WP2*uvr*A at maximal concentrations of 4,980 µg/plate. Additional genotoxicity studies have been conducted on pregabalin and are shown in the Supplementary Table S1 and support a lack of genotoxic activity.

Other experimental data can be evaluated within KC2. Based on an analysis of Tox21 high throughput screening data, at concentration up to 92 μM, pregabalin was negative for induction of ELG1 or p53 and was negative for differential cytotoxicity in the chicken cell DT40 assay using several DNA repair knockout isogenic strains (ICE, 2022). At the concentrations tested in these assays pregabalin was not cytotoxic, indicating that, according to OECD criteria, these tests would not be considered adequate in terms of the maximum concentration tested.

##### 3.1.2.2 *In silico* approaches

**FIGURE 2:**
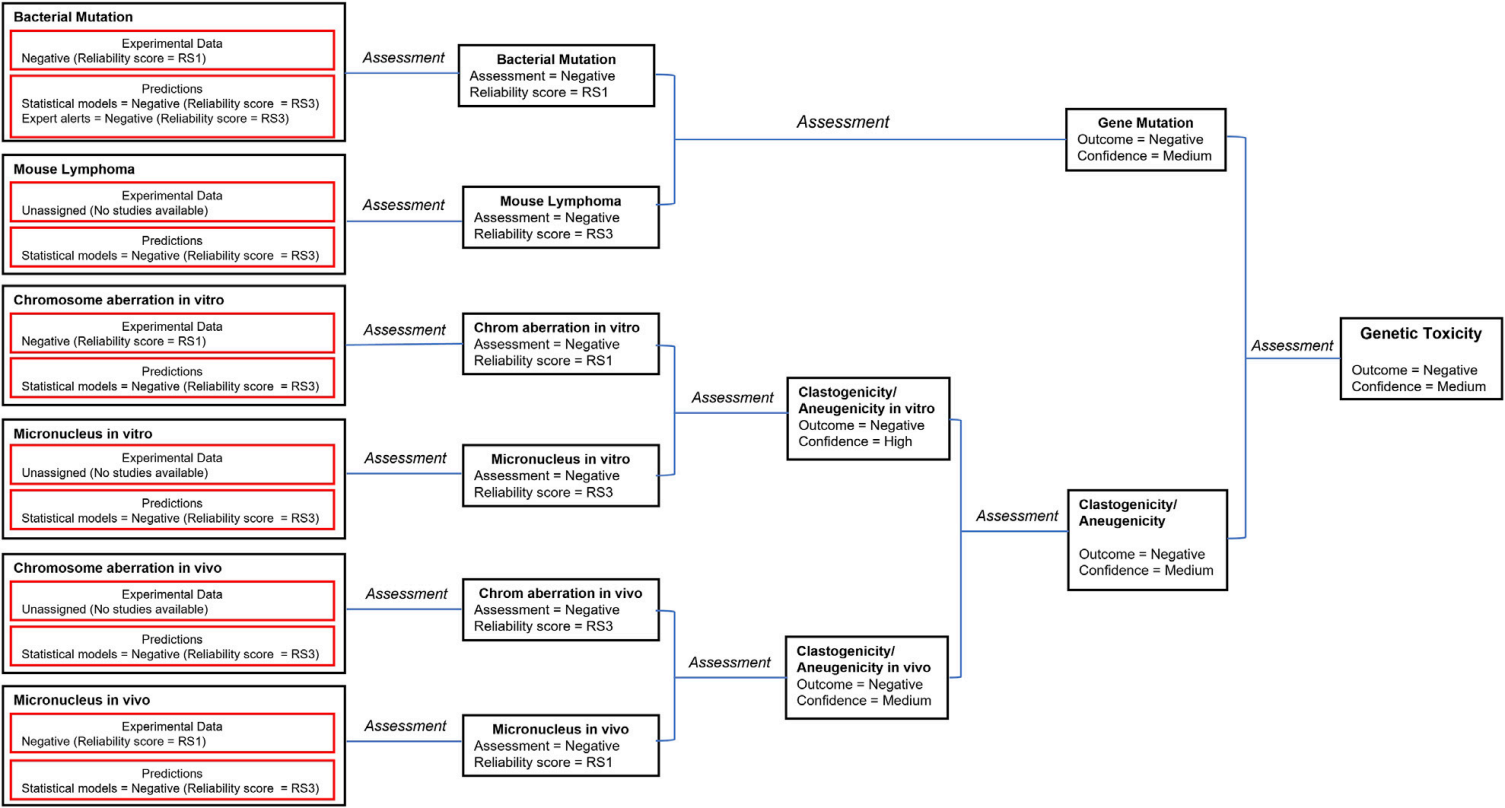
The *in silico* assessments most relevant to genotoxicity are combined with available experimental data according to the genetic toxicology *in silico* protocol (Hasselgren, et al., 2019). Selected models and corresponding reliability scores are shown in the figure. The details of available predictions are reported in the Supplementary Material. The reliability score (RS) of each prediction is documented in the Supplementary Material. This is used to derive the overall endpoint confidence (Myatt, et al., 2018; Johnson, et al., 2022) based on the published rules (Hasselgren, et al., 2019).

A number of *in silico* models provide predictions that relate to genotoxicity including predictions for mutagenicity in bacteria and mouse lymphoma cells as well for the induction of both *in vitro* and *in vivo* chromosomal aberrations and micronuclei (Tice, et al., 2021). The *in silico* predictions leading to the genotoxicity assessment are shown in Figure 2 (the details of the corresponding predictions are reported in Supplementary Table S2). The genetic toxicology *in silico* protocol formulated by Hasselgren and co-workers (Hasselgren, et al., 2019) is used to integrate all of the available information related to genotoxicity. More specifically, the *in silico* predictions are combined with available experimental data from standardized tests to generate an overall endpoint call; in parallel, the corresponding reliability scores of the assessments are used to derive the overall endpoint confidence (Myatt, et al., 2018; Johnson, et al., 2022). Given the lack of significant metabolism for pregabalin in all species tested but the dog, and the knowledge that models and alerts for bacterial mutation based upon parent compounds using the Ames test (with and without metabolic activation) infers assessment of mutation by the metabolites, an analysis of the genotoxicity of possible metabolites was not conducted.

##### 3.1.2.3 Reliability and confidence

The consensus outcome from the integration of the *in silico* genotoxicity models interrogated with the structure of pregabalin was that the compound was negative for genotoxicity (see Figure 2). Based on consideration of both the experimental results and the *in silico* predictions, the overall confidence is high to medium that pregabalin is not genotoxic, and pregabalin is not classified as a genotoxic compound.

##### 3.1.2.4 Data gaps

Although some of the non-regulatory tests were conducted at lower concentrations, given the totality of the data no significant data gaps exist for genotoxicity endpoints.

#### 3.1.3 KC3: Alters DNA repair or causes genomic instability

##### 3.1.3.1 Experimental data

Pregabalin was inactive in the Tox21 DT40 assays that can reflect DNA repair capabilities (ICE, 2022) (Supplementary Table S3). However, this assay does not directly assess DNA repair, so no adequate data are available.

##### 3.1.3.2 In silico approaches

There are no *in silico* methods available for evaluating this endpoint.

##### 3.1.3.3 Reliability and confidence in the data

A confidence score cannot be assigned. Based on available evidence, a robust conclusion on whether pregabalin alters DNA repair or causes genomic instability cannot be derived.

##### 3.1.3.4 Data gaps

Directly relevant studies in mammalian systems are needed to evaluate this KC.

#### 3.1.4 KC4: Induces epigenetic alterations

##### 3.1.4.1 Experimental data

No direct evidence was identified to link pregabalin to an epigenetic mode of action (LYRICA, 2018). Pregabalin functions as an anti-anxiety drug; however, the mode of action is not fully elucidated (LYRICA, 2018). Epigenetic drugs can be used to treat anxiety disorders (Peedicayil, 2020). So, it is possible that pregabalin treats anxiety by epigenetic therapy, but this relationship has yet to be proven. In addition, a number of reviews highlight the role of epigenetic mechanisms in the pathophysiology and treatment of chronic pain (Descalzi, et al., 2015; Geranton and Tochiki, 2015; Liang, et al., 2015). Therefore, although pregabalin is not currently linked to epigenetic mechanisms, there could be a yet undiscovered epigenetic mechanism in line with the therapeutic properties seen in pregabalin. This topic of epigenetic regulation of neurological activity is receiving increased research and regulatory attention (Banik, et al., 2017; EMA, 2018).

However, in contrast to this, Notartomaso et al. (Notartomaso, et al., 2017) studied different painkillers and suggested that pregabalin was not functioning via an epigenetic mechanism. In the study, mice were injected with pregabalin (30 mg/kg) over a series of different experiments. In this study (Notartomaso, et al., 2017) it was specifically chosen to use pregabalin as an active comparator; pregabalin’s known interaction with the α2δ subunit of voltage-sensitive Ca2^+^ channels was used to compare analgesia alongside drugs that enhance acetylation of histones or transcription factors.

There is very little experimental data directly linking pregabalin with any epigenetic interaction or modulation.

##### 3.1.4.2 *In silico* approaches

There are no *in silico* methods for predicting the ability of pregabalin to induce epigenetic alterations.

##### 3.1.4.3 Reliability and confidence

A confidence score cannot be assigned. Based on available evidence, a robust conclusion on whether pregabalin induces epigenetic alterations cannot be derived.

##### 3.1.4.4 Data gaps

Expert literature review found no conclusive link documenting pregabalin with an epigenetic mechanism. As there are no models to address epigenetic modulation directly, this is a gap in our understanding, and, therefore, should be reflected in the confidence of the overall assessment.

#### 3.1.5 KC5: Induces oxidative stress

##### 3.1.5.1 Experimental data

A key event in the mechanism of action for pregabalin carcinogenicity is tissue hypoxia resulting from a sustained alkalosis (Criswell, et al., 2012a; Criswell, et al., 2012b). In mice, chronic tissue hypoxia leads to inflammation (discussed in detail in the next section) characterized by erythrophagocytosis, iron accumulation in macrophages and Kupffer cells, and activated macrophages that release reactive oxygen species (ROS). The inflammation then causes increases in tissue vascular endothelial growth factor (VEGF), platelet-derived growth factor (PDGF), and basic fibroblast growth factor (bFGF) which drive endothelial cell proliferation. Iron deposits in tissues can also lead to increases in ROS. These events do not occur in rats treated with pregabalin (Criswell, et al., 2012a).

Pregabalin was inactive in three p53 assays potentially related to oxidative stress listed in the National Toxicology Program’s Integrated Chemical Environment database (ICE, 2022) (Supplementary Table S3).

##### 3.1.5.2 *In silico* approaches

*In silico* methods are available for some mechanisms that induce oxidative stress (reviewed in (Tice, et al., 2021)). While none were used for pregabalin, models for ROS generation and ARE/Nrf-2 activation could be useful to generate additional understanding of mechanisms involved in carcinogenesis.

##### 3.1.5.3 Reliability and confidence

The experimental data are assigned an overall reliability score of RS2. Given this reliability and consideration of their relevance for the evaluation of oxidative stress, a high confidence can be assigned to the conclusion that pregabalin causes oxidative stress *in vivo*.

##### 3.1.5.4 Data gaps

While the data are considered reliable and relevant, the amount of data on oxidative stress is not large. Additional *in vitro* and *in silico* studies could enhance the understanding of this MOA.

#### 3.1.6 KC6: Induces chronic inflammation

##### 3.1.6.1 Experimental data

Female B6C3F1/CrlBR mice received 1,000 mg/kg bw of pregabalin in the diet for up to 12 months or 5,000 mg/kg bw for up to 29 days (Criswell, et al., 2012c). Dysregulation of angiogenesis and resultant cell death due to chronic hypoxia induced a chronic inflammatory state as evidenced by an increase in activated platelets and an increase in Kupffer cells in the liver and iron-laden macrophages in the bone marrow and spleen in mice, but not in rats. According to the authors (data not shown (Criswell, et al., 2012a),), pregabalin treatment resulted in a dose- and time-dependent increase in activated macrophages in the bone marrow, spleen, and liver in mice–all tissues where hemangiosarcomas were observed. No increases were observed in rats. The authors also reported an increase in the absolute number of white blood cells in treated mice, but the relative distribution of cell types was similar in control and treated animals (data not shown (Criswell, et al., 2012c). Macrophage activation has been shown in other studies to result in cytokine release and subsequent generation of ROS (Corthals, et al., 2006) and platelet activation releases platelet-derived growth factor which is a known chemotactic agent for fibroblasts, vascular smooth muscle cells, and monocytes and can stimulate eosinophils to form superoxide anions (Mannaioni, et al., 1997). In a companion study, addition of vitamin E, an antioxidant, to the mouse diet significantly decreased EC proliferation in mice treated with pregabalin, but not in untreated mice, suggesting that pregabalin treatment was activating EC growth pathways in the mouse, most likely through ROS and inflammation pathways (Criswell, et al., 2012b).

##### 3.1.6.2 *In silico* approaches

While some *in silico* models exist for various parts of inflammatory cascades, these mechanisms are complex and the understanding of factors that promote and sustain the effect are not fully known.

##### 3.1.6.3 Reliability and confidence

The experimental data are overall assigned a reliability score of RS2. Given this reliability and consideration of their corresponding relevance for the evaluation of chronic inflammation, a high to medium confidence can be assigned to the conclusion that pregabalin induces chronic inflammation.

##### 3.1.6.4 Data gaps

Although inflammation is evident with the increase in activated macrophages in liver, spleen, and bone marrow and activated platelets in the peripheral circulation, specific chemical markers of inflammation (i.e., evidence of cytokine/chemokines or myeloperoxidase in the area) were not reported. Furthermore, the presence of ROS was not experimentally verified by direct measurements, although addition of an antioxidant to the diet (vitamin E) provided indirect evidence that these reactive species are required for tumor formation.

#### 3.1.7 KC7: Is immunosuppressive

##### 3.1.7.1 Experimental data

Data on whether pregabalin exerts direct immunomodulatory effects in mammalian systems is mixed, but the weight of evidence suggests it likely has no direct immunosuppressive effects. Minimal to mild effects on the lymphoid system were observed only at very high doses (≥15-fold the human exposure) in rats and at high doses in monkeys. In Health Authority reviews of the data submitted for registration (US)/marketing (EU), dermatopathy on the tail skin of rats and monkeys was noted in nearly all studies; however, skin lesions in other areas were not reported and the effect was not recapitulated in clinical trials. Follow-up studies (LYRICA, 2018) (Pfizer Report 745-03326) (Pfizer Report 250-01888) evaluating the time course of dermatopathy development and its relationship to a wide variety of immune-related endpoints did not support an immune-related cause.

Pregabalin has been investigated in several animal models of disease (Jang, et al., 2012; Hundehege, et al., 2017) in both prophylactic and therapeutic treatment. No effect on the immune responses was observed. Similarly, Silva et al. (Silva, et al., 2014) showed no significant change in the levels of IL-6, IL-10, IL-27 and TGFb in lymph nodes of mice with experimental autoimmune encephalomyelitis that were treated with pregabalin. And while data from a clinical study (Mercan, et al., 2021) seemed to suggest an association between pregabalin treatment and increased immunologic markers in peripheral blood of individuals with neuropathic pain, when confounders such as comorbidities were removed the data did not show any differences.

Of interest, Gao et al. (Gao, et al., 2020) identified the adaptor protein DOK3 as a key regulator microglial cell activation in a model of neuropathic pain. Here, pregabalin was shown to reduce expression of DOK3 mRNA and the induction of inflammatory biomarkers produced by upregulated DOK3, suggesting a role (direct or indirect) on inflammatory responses. In the GLP toxicology studies in mice (Pegg, et al., 2012), there was an increase in the number of macrophages present in bone marrow, spleen, and liver (5-fold greater than controls after 1 year). In a lipopolysaccharide sepsis model in aged rats, Asci et al. (Asci, et al., 2021) showed that pregabalin can inhibit LPS-induced lesions as shown by changes in several immune system markers. The LPS-induced response, however, likely causes the damage through inflammatory processes so no conclusion can be reached from these data regarding direct immunosuppressive effects of pregabalin. Together, these data suggest a possible role for pregabalin in the inflammatory response which is discussed above, though the data are conflicting (e.g., pro-inflammatory in (Pegg, et al., 2012) and anti-inflammatory (Gao, et al., 2020; Asci, et al., 2021)).

##### 3.1.7.2 *In silico* approaches

There are no *in silico* methods for predicting the ability of pregabalin to induce immunomodulatory changes.

##### 3.1.7.3 Reliability and confidence

The data for pregabalin, which are derived from summaries of the original GLP toxicology studies (Pegg, et al., 2012) are assigned a reliability score of RS1. The remainder of the experimental data are assigned a reliability score of RS3. Given these reliability scores and consideration of their corresponding relevance for the evaluation of immunosuppression, a medium confidence can be assigned to the conclusion that pregabalin does not affect immune system function.

##### 3.1.7.4 Data gaps

No other studies were found in the publicly available literature where pregabalin was specifically evaluated for immunosuppressive activity in normal animals (e.g., T-cell-dependent antibody response, assessment of cell-mediated or innate immunity, or evaluation in host resistance models).

#### 3.1.8 KC8: Modulates receptor-mediated effects

##### 3.1.8.1 Experimental data

Criswell et al. (Criswell, et al., 2012b) described the effects of pregabalin treatment in mice on VEGF, PDGF, bFGF, and thrombopoietin (TPO), as well as VEGFR2. There was no increase in serum VEGF or TPO in the study, while serum PDGF increased by 4-fold after 12 months of treatment, and by 47% after 24 months. No increase was observed after 18 months of treatment. Bone marrow and splenic macrophages and erythroid precursor cells were positive for bFGF staining after 6 months of treatment and were strongly positive after 12 months. VEGF levels were increased in spleen at the highest dose level tested after 6 months of treatment, and VEGF was increased in spleen and sternal bone marrow at 1,000 mg/kg after 12 months. No increased VEGF staining was observed in liver. VEGFR2 levels were increased in EC in the liver of female mice at 1,000 mg/kg after 12 months, but not at lower dose levels.

*In vitro*, the only activity in a panel of 182 assays in the ICE database was for estrogen receptor agonist activity, with an AC50 of 11.7 µM (ICE, 2022) (Supplementary Table S3). Pregabalin was considered inactive in the other receptor-mediated assays conducted.

##### 3.1.8.2 *In silico* approaches

The authors of the original studies performed during the development of pregabalin (Criswell, et al., 2012a; Criswell, et al., 2012b; Criswell, et al., 2012c; Pegg, et al., 2012) did not conduct any computational study on their endpoints of interest. Though not necessarily relevant to hemangiosarcoma formation, QSAR models were applied to evaluate androgenic activity, estrogenic (ER) activity, and thyroid peroxidase activity of pregabalin with the Leadscope model applier (Instem, Inc.) and ADMET Predictor (SimulationsPlus) platforms. Such predictions can provide insights on the potential interactions with receptors relevant to other mechanisms of carcinogenicity. Predictions were negative for different endpoints including androgen receptor (AR) binding, aromatase inhibition, thyroperoxidase (TPO) inhibition, thyroid hormone receptor binding and transactivation. Details of the predictions are reported in the Supplementary Table S4.

##### 3.1.8.3 Reliability and confidence

The experimental data are assigned a reliability score of RS2 and standard relevance. The *in silico* results are assigned a RS5. Overall, there is medium confidence that pregabalin does not affect receptor-mediated pathways known to be associated with carcinogenicity.

##### 3.1.8.4 Data gaps

Several computational models have been published for VEGF interactions with VEGFR2 and subsequent proliferation of cells (Mac Gabhann and Popel, 2005; Kleinstreuer, et al., 2013; Clegg and Mac Gabhann, 2015) and use of these models may have strengthened the associations postulated for the mechanism proposed in (Criswell, et al., 2012a; Criswell, et al., 2012c). Similarly, models exist for PDGF activity, and recently a model was reported for PDGF-VEGF interactions with VEGFR2 (Mamer, et al., 2017). However, all of these models are computational biology models and do not predict growth factor activity based on the chemical structure of the binding ligand.

#### 3.1.9 KC9: Causes immortalization

##### 3.1.9.1 Experimental data

No data are available on immortalization of cells exposed to pregabalin, other than the presence of hemangiosarcomas in mice treated with the compound, which implies immortalization of cells.

##### 3.1.9.2 *In silico* approaches

While some *in silico* methods for predicting immortalization in the SHE cell assay are available (Tice, et al., 2021), none were used for pregabalin.

##### 3.1.9.3 Reliability and confidence

Based on a lack of evidence, a robust conclusion on whether pregabalin causes immortalization cannot be derived.

##### 3.1.9.4 Data gaps

Immortalization of cells is typically considered to be an *in vitro* property, relating to the lack of senescence after long-term passaging of cells in culture. Cells taken from malignant tumors usually are immortal when cultured, and non-malignant cells can become immortal in culture when manipulated with certain viruses, proteins, etc., or arise from spontaneous mutations. The utility of *in silico* modeling for immortalization is unknown, as not all immortal cells will progress to malignant tumors.

#### 3.1.10 KC10: Alters cell proliferation, cell death, or nutrient supply

##### 3.1.10.1 Experimental data

Criswell et al. (Criswell, et al., 2012a) described several experiments where EC proliferation was measured in mouse liver, bone marrow, or spleen, and rat liver. Pregabalin increased hepatic endothelial and Kupffer cell proliferation in mice after 12 months of treatment at 200 and 1,000 mg/kg bw, while there was no effect at 50 mg/kg. In another mouse experiment, 5,000 mg/kg bw pregabalin increased the number of proliferating ECs in the liver after 2 and 4 weeks of treatment, and in the bone marrow after 12 weeks. Only minor increases in absolute numbers of proliferating ECs were found in the spleen. Vitamin E supplementation in the diet abolished the EC proliferation in the liver observed after 2 weeks. No increased proliferation of EC was seen in rat liver after up to 18 months of treatment at the maximum tolerated dose. Increases in release of tissue growth factors (VEGF, bFGF, PDGF) could also play a role in proliferation of EC.

No data were found for cell death or nutrient supply.

##### 3.1.10.2 *In silico* approaches

No computational studies were performed on the EC data. As described in Tice and Bassan et al. (Tice, et al., 2021), global *in silico* methods for cell death, cell proliferation, and alteration of nutrient supply are not available.

##### 3.1.10.3 Reliability and confidence

The experimental data are assigned a reliability score of RS2 with standard relevance. Confidence is high to medium that pregabalin affects cell proliferation.

##### 3.1.10.4 Data gaps

The understanding of the mechanistic drivers for proliferation of EC are incomplete, and *in silico* models for the processes involved in KC10 are not available.

### 3.2 Other data related to carcinogenicity

A ChEMBL search retrieved no evidence of activity at targets related to known mechanisms of carcinogenicity. For pregabalin itself, no pChEMBL values were found other than for its recognized target: voltage-dependent calcium channels, α2δ subunit. A search for compounds with ≥50% structural similarity to pregabalin retrieved only two compounds (50% and 53% similar to pregabalin), both with only very low potencies (>30 µM) at four targets: CYP1A2, putative fructose-1,6-bisphosphate aldolase, carbonic anhydrase II, and solute carrier family 22 member 20.

Data for pregabalin in the Comparative Toxicogenomics Database (CTDB, 2022) shows gene interactions with angiotensin-related receptors and a few other genes that have no direct relationship to carcinogenesis.

A safety signal analysis of spontaneous reporting systems was performed with the ClarityPV platform (https://claritypv.com/) (CLARITY, 2023). The analysis was based on a total number of 397,205 unique spontaneous reports deposited between 1 January 2005 and 31 August 2023 (average reporting rate of 1,773 reports/month or 21,278 reports/year) in FAERS (250,457), VigiBase (130,039), JADER (13,973) and VAERS (2,736). A list of 22 side effects disproportionally reported (PRR05 > 2.0) for pregabalin was identified (Supplementary Table S5), of which pituitary tumour benign shows also levels of suspiciousness and instantiation that warn some cautionary vigilance. Pregabalin was first approved for marketing in the US in 2004 and in the EU in 2005. Of note, some patients treated with pregabalin may also have been treated with dopamine-active agents, which are associated with pituitary tumors.

### 3.3 Carcinogenicity models and their predictions

The *in silico* models available for predicting *in vivo* carcinogenicity are based on resources that collect carcinogenicity results from the corresponding animal studies (Benigni, et al., 2008; Golbamaki and Benfenati, 2016; Bossa, et al., 2018; Bower, et al., 2020). The specific carcinogenicity predictions for pregabalin are reported in Table 2, where detailed results are included for both statistical- and expert-based systems (i.e., structural alerts). No alerts for both the genotoxic and the non-genotoxic carcinogenicity mechanisms are reported and, similarly, the statistical-based predictions falling in the applicability domain of the corresponding models (as in the case of the *in vivo* rodent carcinogenicity models for female rat, male rat, female mouse and male mouse (Instem, 2022)) are negative. There are also models providing a quantitative prediction (TD50); however, these models generally have a limited predicting capacity. Two of these software programs, ADMET Predictor and LAZAR, predict carcinogenicity potency expressed as TD50 (the oral daily dose administered over the course of lifetime required to produce tumors in 50 percent of animals); these values were compared with those reported by Pegg, et al. (2012). Both ADMET predictor and Lazar are statistically-based models that use Carcinogenicity Potency Database (CPDB, 2022) data to model endpoints of interest. In the case of Lazar prediction for rat carcinogenicity, the confidence in the prediction was considered low by the program. Comparison of the predictions by ADMET predictor (predicted TD50 rat = 92.2 mg/kg/day and predicted TD50 mouse = 376.8 mg/kg/day) to the bioassay results shows the *in silico* predictions to be far away from the actual TD50 of >5,000 mg/kg in the mouse. The reason for ADMET prediction of pregabalin being more potent for rat carcinogenicity than mouse is not clear. Overall, the *in silico* outcome for carcinogenicity for pregabalin *in vivo* does not highlight any element of concern. However, reliability scores of the predictions (Myatt, et al., 2018; Johnson, et al., 2022) as evaluated by means of analysis and expert review of the results are not high (mostly RS5) and this lowers the confidence of the negative overall assessment for *in vivo* carcinogenicity. The models did not predict the experimental outcome in the mouse, indicating that while they were correct in predicting a low likelihood of carcinogenicity with pregabalin across species, consideration of mechanisms of carcinogenicity is not a strength of these models.

**TABLE 2:** Carcinogenicity predictions for pregabalin.

Tool	Endpoint	Model	Data/Prediction^a^	Applicability domain	Call	Comments
Leadscope Model Applier (v. 3.1.0-40)	Carcinogenicity *in vivo*	Carc female mouse v3	Negative (PPP = 0.13)	In domain	Negative	1) Low positive prediction probability provided by the statistical model (PPP = 0.13)
						2) The identified model features are mainly represented in experimentally negative compounds and the identified negative features provide a higher contribution to the result, resulting in an overall negative prediction call (PPP = 0.13). However, the structure of the target molecule is not fully covered by the features used to derive the prediction, i.e., the methylamine moiety is not covered
						3) No relevant training set analogs, meaning that the target molecule is only limited represented in the training set.
						4) Concordance of the analogs: the mostly similar training analog, i.e., Gabapentin, has positive experimental data in disagreement with the prediction
						5) Prediction accuracy of the analogs: the mostly similar training analog, i.e., Gabapentin, is not correctly predicted by the model introducing an uncertainty in the prediction derived for the target molecule
						6) Based on the poor coverage of the structure of the target molecule and the not optimal concordance and accuracy of the mostly similar training analog, the reliability score cannot be upgraded from the default RS5
		Carc male mouse v3	Negative (PPP = 0.223)	In domain	Negative	1) Low positive prediction probability provided by the statistical model (PPP = 0.223)
						2) The identified model features provide a good coverage of the structure; they are mainly represented in experimentally negative compounds and the identified negative features provide a higher contribution to the result, resulting in an overall negative prediction call (PPP = 0.223)
						3) No relevant training set analogs, meaning that the target molecule is only limited represented in the training set.
						4) Concordance of the analogs: the mostly similar training analog, i.e., Gabapentin, has positive experimental data in disagreement with the prediction
						5) Prediction accuracy of the analogs: the mostly similar training analog, i.e., Gabapentin, is not correctly predicted by the model introducing an uncertainty in the prediction derived for the target molecule
						6) Based on the not optimal concordance and accuracy of the mostly similar training analog, the reliability score cannot be upgraded from the default RS5
		Carc male rat v3	NEGATIVE (PPP = 0.0987)	In domain	Negative	1) Low positive prediction probability provided by the statistical model (PPP = 0.0987)
						2) The identified model features provide a good coverage of the structure; they are mainly represented in experimentally negative compounds and the identified negative features provide a higher contribution to the result, resulting in an overall negative prediction call (PPP = 0.0987)
						3) No relevant training set analogs, meaning that the target molecule is only limited represented in the training set.
						4) Concordance of the analogs: the mostly similar training analog, i.e., Gabapentin, has positive experimental data in disagreement with the prediction
						5) Prediction accuracy of the analogs: the mostly similar training analog, i.e., Gabapentin, is not correctly predicted by the model introducing an uncertainty in the prediction derived for the target molecule
						6) Based on the not optimal concordance and accuracy of the mostly similar training analog, the reliability score cannot be upgraded from the default RS5
		Carc female rat v3	Negative (PPP = 0.161)	In domain	Negative	1) Low positive prediction probability is provided by the statistical model (PPP = 0.161), meaning that the target molecule is predicted as negative
						2) The identified model features provide a good coverage of the structure; they are mainly represented in experimentally negative compounds and the identified negative features provide a higher contribution to the result, resulting in an overall clear negative prediction call (PPP = 0.161)
						3) Training set analogs were inspected and no concern arose by this analysis. Analogs are characterized by a limited structural similarity with respect to the target molecule, meaning that the target molecule is only limited represented in the training set.
						4) The mostly similar analog is Gabapentin, which is experimentally negative and correctly predicted by the model
						The reliability score is then upgraded to RS3
Derek Nexus: 6.1.0, Nexus: 2.3.0	Carcinogenicity	Expert alerts	No alerts fired	Not applicable	Not assigned	No alerts associated with carcinogenicity are fired by the expert system. Because of the nature of the model, this is not a negative prediction. It, however, supports any negative result from other model(s)
VEGA (v. 1.3.10)	Carcinogenicity *in vivo*	Carcinogenicity model (CAESAR) 2.1.10	Positive	Outside the applicability domain (AD = 0)	Rejected	This prediction is rejected given its low reliability
		Carcinogenicity model (ISS) 1.0.3	Negative	Outside the applicability domain (AD = 0)	Rejected	This prediction is rejected given its low reliability
		Carcinogenicity model (IRFMN-ISSCAN-CGX) 1.0.1	Possible NON-Carcinogen	Outside the applicability domain (AD = 0.52)	Rejected	This prediction is rejected given its low reliability
		Carcinogenicity model (IRFMN-Antares) 1.0.1	Possible NON-Carcinogen	Outside the applicability domain (AD = 0.555)	Rejected	This prediction is rejected given its low reliability
		Carcinogenicity oral classification model (IRFMN) 1.0.1	Carcinogen	The predicted compound could be out of the Applicability Domain of the model (AD = 0.754)	Rejected	This prediction is rejected given its low reliability
		Carcinogenicity in male rat (CORAL) 1.0.0	Predicted TD50 [mg/kg bw/day]: 2.48	Outside the applicability domain	Rejected	This prediction is rejected given its low reliability
		Carcinogenicity in female Rat (CORAL) 1.0.0	Predicted TD50 [mg/kg bw/day]: 8588.44	Outside the applicability domain	Rejected	This prediction is rejected given its low reliability
Toxtree v 3.1.0	Carcinogenicity	Genotoxic and/or non-genotoxic carcinogenicity alerts by ISS	No alerts fired (Negative for genotoxic carcinogenicity and Negative for nongenotoxic carcinogenicity)	Not available	Not assigned	This model is also available in the OECD QSAR Toolbox
LAZAR (v. 1.4.2)	Carcinogenicity *in vivo*	Carcinogenicity (Mouse (TD50))	-	Out of domain	-	-
		Carcinogenicity (Rat (TD50))	2560.0 (mg/kg_bw/day)	Low confidence (Insufficient number of neighbors for regression model, using weighted average of similar substances)	-	-

^a^ The predictions may be associated with statistical value such as the PPP, that is the positive prediction probability (the positive prediction probability is given as the likelihood value between 0 (non-toxic) and 1 (toxic)).

### 3.4 Species differences/human relevance

Pregabalin treatment for up to 2 years caused hemangiosarcoma formation in mice, but not in rats (Pegg, et al., 2012). The mode of action of pregabalin-induced hemangiosarcomas is formulated in (Criswell, et al., 2012a; Criswell, et al., 2012b; Pegg, et al., 2012). The data and conclusions in these publications are consistent with the mode of action for hemangiosarcoma formation described in (Cohen, 2017). Criswell et al. (Criswell, et al., 2012c) further describe the relevance of mouse hemangiosarcomas to humans, including some data from studies with human blood or cells, and *in vivo* from human subjects. Regarding human relevance of results from rodent studies, it is recognized that tumors observed in animal studies that result from genotoxic mechanisms are generally considered to be relevant to humans even when occurring in tissues with no direct human equivalent (ECHA, 2017). On the other hand, non-genotoxic compounds causing tumors in animals may act through modes of action that are not human relevant (ECHA, 2017; Goodman, 2018). The available data point to a lack of relevance of the pregabalin-induced mouse hemangiosarcomas to humans.

## 4 Discussion

Lifetime rodent carcinogenicity studies are extremely resource intensive, requiring the use of over 500 rodents, costing over $1 million and taking approximately 3 years of time to complete. As it is not possible or desirable to test all chemicals and drugs under these conditions, the development and use of *in vitro* and *in silico* tools to predict carcinogenicity is imperative. Tice and Bassan et al. (Tice, et al., 2021) described the state-of-the-art for the use of *in silico* tools to predict the outcome of *in vitro* and *in vivo* assays (other than the traditional rodent carcinogenicity assay) relevant to carcinogenicity hazard assessment. Using pregabalin as a case study, we reviewed the *in vivo*, *in vitro*, and *in silico* data organized within the framework of the 10 KCs of carcinogens. We show where the experimental and *in silico* models give results that are useful in predicting carcinogenicity, and where there are gaps in the data and models that need to be addressed to more reliably predict nongenotoxic compound carcinogenicity.

**FIGURE 3:**
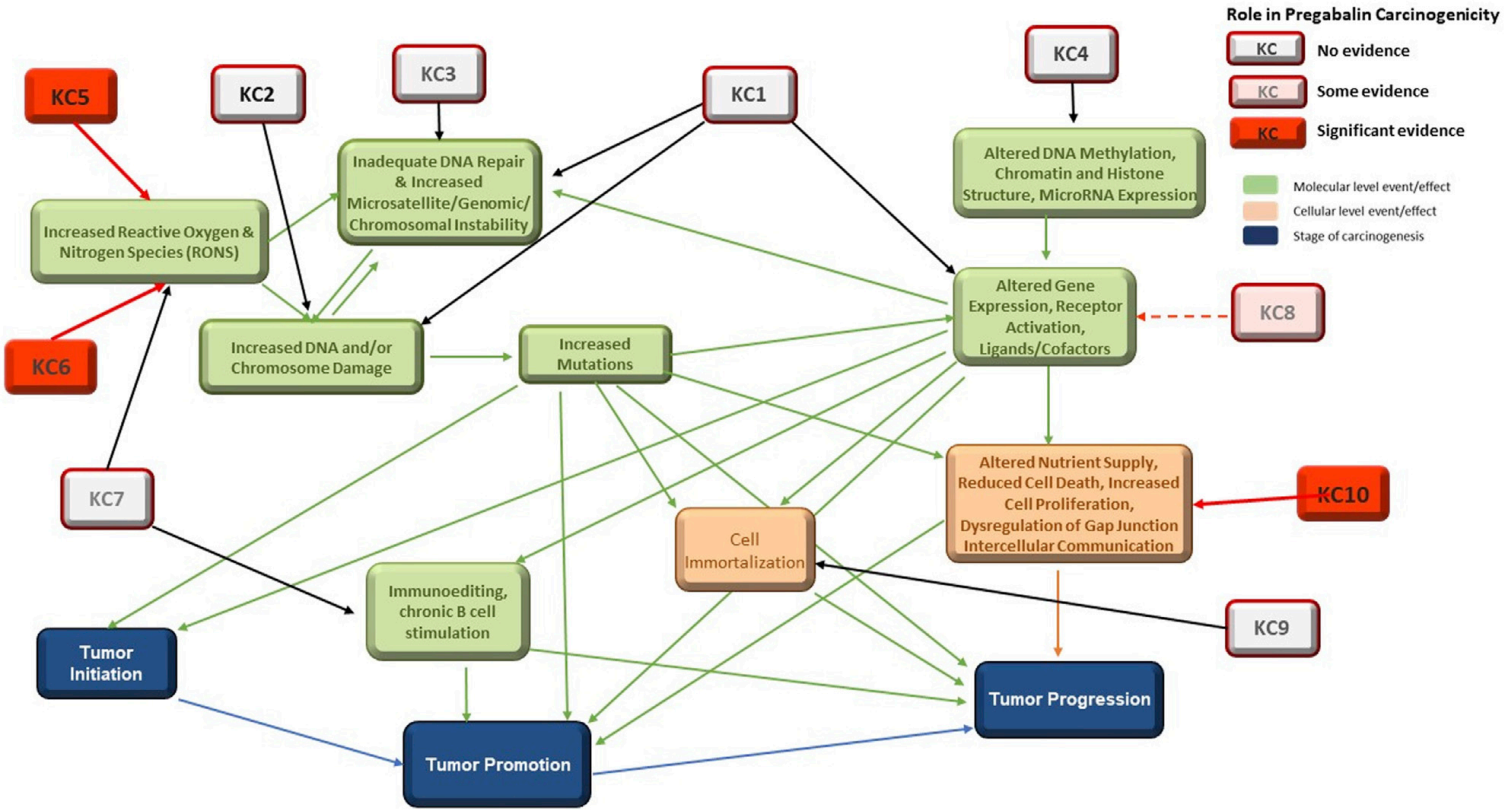
Interactions among the 10 KCs and with the Three Stages of Carcinogenesis. KC1 = is electrophilic, KC2 = is genotoxic, KC3 = alters DNA repair or causes genomic instability, KC4 = induces epigenetic alterations, KC5 = induces oxidative stress, KC6 = induces chronic inflammation, KC7 = is immunosuppressive, KC8 = modulates receptor-mediated effects, KC9 = causes immortalization, KC10 = alters cell proliferation, cell death, or nutrient supply. Specific evidence for involvement in pregabalin carcinogenicity is highlighted in red.

Pregabalin is a single species, nongenotoxic rodent carcinogen. The MOA for pregabalin carcinogenicity has been proposed by (Criswell, et al., 2012c) and has been accepted by regulators globally. This MOA is consistent with the MOA of other agents that cause hemangiosarcomas in rodents (Cohen, 2017). Figure 1 in (Tice, et al., 2021) showed the relationship between the KCs of carcinogens and the stages of carcinogenesis. Here, the figure has been revised to highlight the roles of the KCs involved in the carcinogenic process for pregabalin (Figure 3).

Outcomes for KC1 (is electrophilic or can be metabolically activates), KC2 (is genotoxic), and KC8 (modulates receptor-mediated effects) can, at least in part, be reliably predicted with *in vitro* systems and *in silico* models (Table 3). These are discussed in detail in (Tice, et al., 2021). While models to predict electrophilicity may be overly sensitive, bacterial mutagenicity models that incorporate metabolic activation are an acceptable and more accurate surrogate for the electrophilicity endpoint. Prediction of KC2 (genotoxicity) is the most well-developed area of *in vitro* and *in silico* tools for carcinogenicity assessment and *in vivo* tests are rarely needed. Prediction of KC8 outcomes (modulates receptor-mediated effects) was applied in this work for some nuclear receptor activities, with the most effort centered on ER and AR which have been linked to certain mechanisms of carcinogenicity. Interactions with some additional receptors, CYPs and AhR can be modeled (Vedani, et al., 2012). While these activities are not part of the pregabalin carcinogenicity MOA, they could play a role in the carcinogenicity of endocrine-disrupting chemicals (Heusinkveld, et al., 2020). Some receptor-mediated mechanisms of carcinogenicity, such as PPARɑ and AhR driven tumors, could lack relevance to humans, which highlights the need for species-specific understanding of mechanisms and models.

**TABLE 3:** Summary of data reliability and confidence. Reliability scores and confidence levels are assigned according to (Myatt, et al., 2018; Johnson, et al., 2022). Confidence considers the reliability, relevance, and coverage of information available. KCs listed in red are involved in the mode of action of pregabalin carcinogenicity in mice.

Key characteristic	Reliability		Confidence
	Experimental	In Silico	
KC1: Is Electrophilic	RS1	RS3	Medium
KC2: Is Genotoxic	RS1	RS1-RS3	Medium to High
KC3: Alters DNA Repair or Causes Genomic Instability	^a^	^a^	No Confidence
KC4: Induces Epigenetic Changes	^a^	^a^	No Confidence
KC5: Induces Oxidative Stress	RS2	^a^	High
KC6: Induces Chronic Inflammation	RS2	^a^	Medium to High
KC7: Is Immunosuppressive	RS1-RS3	^a^	Medium
KC8: Modulates Receptor-Mediated Effects	RS2	RS5	Medium
KC9: Causes Immortalization	^a^	^a^	No Confidence
KC10: Induces Cell Proliferation, Cell Death, Nutrient Supply	RS2	^a^	Medium to High

^a^ Insufficient data to make assignment.

A variety of *in vitro* systems are available for prediction of KC5 (induce oxidative stress), but *in silico* model development has lagged. As described in (Tice, et al., 2021) *in silico* models are available for several hard chemistry endpoints related to oxidative stress, such as peroxide and quinone formation. Of note, a quantum model for Nrf-2/ARE activation has been reported in the literature to identify the structures predicted to activate the Nrf2-antioxidant response element pathways (Williamson, et al., 2012). As oxidative stress plays a role in the MOA for pregabalin carcinogenicity, a readily available model for the prediction of these types of effects would be desirable.

In contrast, no significant *in silico* models exist for prediction of KC3 (alerts DNA repair or causes genomic instability), 4 (induces epigenetic alterations), 6 (induces chronic inflammation), 7 (is immunosuppressive), 9 (causes immortalization), and 10 (alters cell proliferation, cell death, or nutrient supply). *In silico* models exist for only a small number of the endocrine and hormonal endpoints that can be associated with carcinogenicity. *In vivo* and *in vitro* assays are available for some aspects of these KCs, but prediction from the chemical structure of the molecule (i.e., drug, xenobiotics) is not possible at this time. This is a major obstacle for prediction of nongenotoxic carcinogenicity, as the MOA is often dependent on KC6 (inflammation) and/or KC10 (cell proliferation). DNA repair (KC3) and epigenetic factors (KC4 (Shin, 2020)) could play significant roles in carcinogenicity of agents that are not positive in traditional genotoxicity assays (KC2). While many immunosuppressive drugs carry a warning for increased cancer risk (Cangemi, et al., 2019), the extent of immunosuppression associated with this risk in humans is unknown. Additional *in vitro* assays and *in silico* models to predict these effects would be extremely useful.

Data from other studies such as Tox21-type screening, toxicogenomics, target activity profiles such as ChEMBL, and human data, when available, can add value to carcinogenicity assessments, but *in silico* models are not available for any of these. Cook et al. (Cook, et al., 2018) investigated three additional, structurally diverse compounds that caused hemangiosarcomas in mice (fenretinide, troglitazone, and elmiron), further testing the MOA proposed in (Cohen, et al., 2009; Criswell, et al., 2012c). These studies included some of the same analyses used in the pregabalin studies (bone marrow, hematology, and hypoxia parameters) as well as transcriptomics. The results indicated that the three additional compounds initiated the same MOA as pregabalin, with the potency of biological effects following the potency of hemangiosarcoma formation by these compounds. Additionally, the studies showed that transcriptomics were consistent with the MOA and potency of the compounds but was not more sensitive than hydroxyprobe for detection of hypoxia. Given the structural diversity of compounds that cause hemangiosarcomas in mice, the availability of *in silico* models to predict some of the key elements of this MOA could save significant amounts of effort, time, and animals. The use of *in silico* models to predict hazard for the different KC could be very useful in determining what targeted biological assays to perform to confirm an effect.

While known human carcinogens are almost exclusively genotoxic compounds, nongenotoxic carcinogenicity has been shown in experimental systems and in most cases the relevance to humans is not known with any degree of confidence (Silva Lima and van der Laan, 2000; Cohen, 2017). This is the case for environmental chemicals, food source chemicals, and drugs, highlighting the need for reliable methods for predicting the key events in an MOA, and KCs of carcinogens can be a useful method as an initial step to organize and process the data. As an example of where reliable *in silico* methods for prediction of nongenotoxic carcinogens would be of use, the ICH has revised the S1B guideline to allow for drug developers to develop a weight of evidence (WoE) argument to assess whether a rat carcinogenicity study would add value over existing data for determination of human carcinogenicity. The proposed WoE assessment (ICH, 2022) combines certain factors such as target biology, genotoxicity, secondary pharmacology, immunomodulation, hormonal perturbation, and repeated-dose histopathology into an integrated human risk assessment. The KCs framework may be one approach to support the identification and interpretation of relevant evidence and assays for each factor supporting how such evidence might be combined, and relevant *in silico* predictions would provide additional insights into the ICH S1B weight of evidence approach. This can be particularly useful when a specific MOA is not postulated.

## 5 Conclusion

The overall goal of this exercise was to evaluate the ability of *in silico* models to predict nongenotoxic carcinogenicity with pregabalin as a case study while being guided by the KC framework in the organization and combination of the collected information. Pregabalin is a single-species carcinogen producing only one type of tumor, hemangiosarcomas. The established MOA is triggered by tissue hypoxia, leading to oxidative stress (KC5), chronic inflammation (KC6), and increased cell proliferation (KC10) of EC (Criswell, et al., 2012a). Of these KCs, *in silico* models are available only for selected endpoints in KC5, limiting the usefulness of computational tools in prediction of pregabalin carcinogenicity. KC1, KC2, and KC8, for which predictive *in silico* models exist, do not play a role in this MOA. Additionally, as the pregabalin MOA is considered not relevant to humans, experimental assays and *in silico* models used to predict endpoints for the KC involved must either account for species differences or produce results that can be interpreted in the context of species-specific biology.

We investigated the availability of *in silico* models to predict the ten KCs of carcinogens for a nongenotoxic compound, pregabalin. *In silico* approaches are available for some of the mechanisms associated with the KCs but are particularly lacking for the KCs involved in the MOA specific for pregabalin carcinogenicity. Development of reliable *in silico* models for prediction of oxidative stress, chronic inflammation, immunosuppression, and cell proliferation will be critical for the ability to predict nongenotoxic compound carcinogenicity.

## Data Availability

The original contributions presented in the study are included in the article/Supplementary Material, further inquiries can be directed to the corresponding author.

## Abbreviations

bFGF, basic fibroblast growth factor; EC, Endothelial cell; GLP, Good laboratory practices; ICH, International Council for Harmonisation of Technical Requirements for Pharmaceuticals for Human Use; IST, In silico toxicology protocol; KC, Key characteristic; MOA, Mode of action; OECD, Organisation for Economic Cooperation and Development; PDGF, Platelet-derived growth factor (Q)SAR, Quantitative structure-activity relationship; ROS, Reactive oxygen species; RS, Reliability score; TD50, Toxic dose 50; TPO, Thrombopoietin; VDCC, voltage-dependent calcium channel; VEGF, Vascular endothelial growth factor.
