# Supplementary material for: In silico approaches in carcinogenicity hazard assessment: case study of pregabalin, a nongenotoxic mouse carcinogen
Source: Front Toxicol. 2023 Nov 13;5:1234498. doi: 10.3389/ftox.2023.1234498 (PMC10679394; doi:10.3389/ftox.2023.1234498)
Supplement: Supplementary file 1 [file Table1.docx]

# Supplemental Data

## Table S1: Genotoxicity Assay Results for Pregabalin

| Assay | Test System | Mode of Administration | Outcome |
| --- | --- | --- | --- |
| Bacterial mutagenicity^1^ | *Salmonella typhimurium* | In vitro | Negative |
| Bacterial mutagenicity^1^ | *Escherichia coli* | In vitro | Negative |
| Point Mutation^1^ | Chinese hamster ovary cells | In vitro | Negative |
| Sister Chromatid Exchange^1^ | Chinese hamster ovary cells | In vitro | Negative |
| Unscheduled DNA Synthesis^1^ | Mouse, Rat | In vivo | Negative |
| Micronucleus^1^ | Mouse, Rat | In vivo | Negative |
| Somatic Mutation and Recombination Test^2^ | *Drosophila melanogaster* | In vivo | Weakly active |

^1^ From Pegg et al., 2012.
^2^ From Yüksel et al., 2010.

## Table S2a. Bacterial mutation predictions for pregabalin. The available results support a negative call for the bacterial mutation endpoint with a reliability score^[[1]](#footnote-1)^ of RS3. If the experimental value is taken into account, a reliability score of RS1 can be assigned. Similar results are obtained for the metabolite, N-methylpregabalin.

| **Tool** | **Endpoint** | **Model** | **Data/**  **Prediction^§^** | **Applicability domain** | **Call** | **Comments** |
| --- | --- | --- | --- | --- | --- | --- |
| Leadscope Model Applier  (v. 3.1.0-40) | Bacterial mutation | Experimental data | Negative | - | Negative | The prediction results have been reviewed alongside the available experimental data and a reliability score has been assigned to the overall assessment* |
|  |  | QSAR model v2 | Negative  (PPP = 0.118) | In domain |  |  |
|  |  | Expert alerts v8 | Negative | In domain |  |  |
| Derek Nexus: 6.1.0, Nexus: 2.3.0** | Bacterial mutation | Expert alerts | Inactive | No misclassified or unclassified features | Negative | The absence of misclassified or unclassified features is indicative of a robust prediction. |
| EPA T.E.S.T. Version 5.1.1 | Bacterial mutation (*Salmonella typhimurium*) | Consensus method | Negative (predicted value = 0.29) | In domain | Negative | The prediction is in domain and similar compounds are available supporting the negative prediction. |
| VEGA  (v. 1.2.8) | Bacterial mutation | Ames test CONSENSUS model  v. 1.0.3 | Non-Mutagenic with a consensus score of 0.825 | In domain | Negative | The negative prediction is a consensus from 4 different models giving a negative result with high (three models) and moderate (one model) reliability. In particular, the prediction by the KNN/Read-across model has some critical aspects (the prediction may be out of domain). However, overall, the negative outcome of the consensus model can be considered robust. |
| ToxTree  (v. 3.1.0) | Bacterial mutation (*Salmonella typhimurium*) | In vitro mutagenicity (Ames test) alerts by ISS | No alerts found for *S. Typhimurium* | N/A | Negative | This model is also made available by the OECD QSAR Toolbox. |

§ The predictions may be associated with statistical value such as the PPP that is the positive prediction probability (the positive prediction probability is given as the likelihood value between 0 (non-toxic) and 1 (toxic)).

#### *Expert review: Leadscope bacterial mutation prediction

1) Pregabalin is reported as not mutagenic in bacterial *in vitro* tests compliant with the OECD 471 five strains requirement in the Leadscope (Instem) database^[[2]](#footnote-2)^.

2) The negative predictions given by the statistical and expert rule-based model are driven by the correspondence of target molecule with an experimentally negative training/reference compound.

3) No structural alert is identified by the expert rule-based system.

4) Low positive prediction probability is provided by the statistical model (PPP = 0.118), meaning that, excluding the experimental data, the target molecule is predicted as clearly negative.

5) The identified model features provide a good coverage of the structure; they are mainly represented in experimentally negative compounds and the identified negative features provide a higher contribution to the result, resulting in an overall clear negative prediction call (PPP= 0.118).

6) Training/reference set analogs were inspected, and no concern arose by this analysis. Analogs are characterized by a good structural similarity with respect to the target molecule, meaning that the target molecule is well represented in the training/alert reference set.

7) Good concordance of the analogs: the analogs have negative experimental Ames data in agreement with the prediction.

8) Good prediction accuracy of the analogs: the analogs are correctly predicted as negative confirming the good performance of the model on these type of molecules.

9) In addition to training/reference alert set analogs, 4-Aminobutyric acid is also identified as relevant compound which is experimentally negative as documented on ECHA website.^^[[3]](#footnote-3)^^

** It is noted here that Derek Chromosome damage, Mutagenicity *in vivo,* Nonspecific genotoxicity

## Table S2b. Mouse Lymphoma predictions for pregabalin. The available results support a negative call for the mouse lymphoma endpoint with a reliability score^1^ of RS3.

| **Tool** | **Endpoint** | **Model** | **Data/**  **Prediction**^§^ | **Applicability domain** | **Call** | **Comments** |
| --- | --- | --- | --- | --- | --- | --- |
| Leadscope Model Applier  (v. 3.1.0-40) | Mouse Lymphoma | Leadscope Mouse Lymphoma Act v2 | Negative  (PPP = 0.0725) | In domain | Negative | The prediction results have been reviewed; accordingly, a reliability score has been assigned to the overall assessment* |
|  |  | Leadscope Mouse Lymphoma Unact v2 | Negative  (PPP = 0.0421) | In domain |  |  |

§ The predictions may be associated with statistical value such as the PPP that is the positive prediction probability (the positive prediction probability is given as the likelihood value between 0 (non-toxic) and 1 (toxic)).

#### * Expert review: Leadscope Mouse Lymphoma

1) Low positive prediction probability provided by the Mouse Lymphoma Act and Mouse Lymphoma Unact model (PPP = 0.0725 and PPP = 0.0421).

2) The identified model features provide a good coverage of the structure; they are mainly represented in experimentally negative compounds and the identified negative features provide a higher contribution to the result, resulting in an overall negative prediction calls (PPP= 0.0725 and PPP = 0.0421).

3) Training set analogs were inspected and no concern arose by this analysis. Analogs are characterized by a limited structural similarity with respect to the target molecule, meaning that the target molecule is only limited represented in the training set.

## Table S2c. Chromosome Aberration (*in vitro/in vivo*) predictions for pregabalin. The available results support a negative call for the chromosome aberration *in vitro* endpoint with a reliability score^1^ of RS3; if the experimental value is taken into account, a reliability score of RS1 can be assigned. The available results support a negative call for the Chromosome Aberration *in vivo* endpoint with a reliability score of RS3.

| **Tool** | **Endpoint** | **Model** | **Data/**  **Prediction**^§^ | **Applicability domain** | **Call** | **Comments** |
| --- | --- | --- | --- | --- | --- | --- |
| Leadscope Model Applier  (v. 3.1.0-40) | Chromosome Aberration in vitro | Experimental data | Negative | - | Negative | The prediction results have been reviewed; accordingly, a reliability score has been assigned to the overall assessment* |
|  |  | Leadscope In Vitro Chrom Ab CHL v2 | Negative  (PPP = 0.215) | In domain |  |  |
| VEGA  (v. 1.2.8) | Chromosomal aberration activity in vitro | (CORAL) v. 1.0.0 | Inactive | In domain (ADI = 0.924) | Negative | The prediction is highly reliable. Available analogues support the result. |
| Derek Nexus: 6.1.0, Nexus: 2.3.0 | Chromosome damage in vitro | Expert alerts | No alerts fired | Not applicable | Not assigned | No alerts associated with Chromosome damage in vitro are fired. Because of the nature of the model, this is not a negative prediction. It, however, supports any negative result from other model(s). |
| Leadscope Model Applier  (v. 3.1.0-40) | Chromosome Aberrations in vivo | Leadscope In Vivo Chrom Ab Comp v2 | Negative  (PPP = 0.021) | In domain | Negative | The prediction results have been reviewed; accordingly, a reliability score has been assigned to the overall assessment** |
| Derek Nexus: 6.1.0, Nexus: 2.3.0 | Chromosome damage in vivo | Expert alerts | No alerts fired | Not applicable | Not assigned | No alerts associated with Chromosome damage in vivo are fired. Because of the nature of the model this is not a negative prediction, rather a lack of evidence of activity. It, however, supports any negative result from other model(s). |
| OECD QSAR Toolbox 4.5 | Chromosomal aberration | Protein binding alerts for Chromosomal aberration by OASIS | No alerts fired | Not applicable | Not assigned | This model is a profiler that does not provide additional elements for an expert analysis. |

§ The predictions may be associated with a statistical value such as the PPP that is the positive prediction probability (the positive prediction probability is given as the likelihood value between 0 (non-toxic) and 1 (toxic)).

#### * Expert review: Leadscope Chromosome Aberration in vitro

1) Pregabalin is reported as to be negative in valid *in vitro* chromosome aberration test (Chinese hamster)^2^.

2) Low positive prediction probability is provided by the statistical model (PPP = 0.215), meaning that the target molecule is predicted as negative.

3) The identified model features provide a good coverage of the structure; they are mainly represented in experimentally negative compounds and the identified negative features provide a higher contribution to the result, resulting in an overall clear negative prediction call (PPP= 0.215).

4) Training set analogs were inspected, and no concern arose by this analysis. Analogs are characterized by a limited structural similarity with respect to the target molecule, meaning that the target molecule is only limited represented in the training set.

5) The mostly similar analogs are Gabapentin and Glutamic acid, which are experimentally negative and correctly predicted by the model.

#### ** Expert review: Leadscope Chromosome Aberration in vivo

1) Low positive prediction probability provided by the In Vivo Chrom Ab Comp model (PPP = 0.021).

2) The identified model features provide a good coverage of the structure; they are mainly represented in experimentally negative compounds and the identified negative features provide a higher contribution to the result, resulting in an overall negative prediction call (PPP= 0.021).

3) Training set analogs were inspected and no concern arose by this analysis. Analogs are characterized by a limited structural similarity with respect to the target molecule, meaning that the target molecule is only limited represented in the training set.

## Table S2d. Micronucleus (*in vivo/in vitro*) predictions for pregabalin. The available results (see table below) support a negative call for the micronucleus *in vivo* endpoint with a reliability score ^1^ of RS3; if the experimental value is taken into account, a reliability score of RS1 can be assigned. The available results (see table below) support a negative call for the micronucleus *in vitro* endpoint with a reliability score of RS3.

| **Tool** | **Endpoint** | **Model** | **Data/**  **Prediction**^§^ | **Applicability domain** | **Call** | **Comments** |
| --- | --- | --- | --- | --- | --- | --- |
| Leadscope Model Applier  (v. 3.1.0-40) | Micronucleus In vivo | Experimental data | Negative | - | Negative | The prediction results have been reviewed; accordingly, a reliability score has been assigned to the overall assessment* |
|  |  | Leadscope In Vivo Micronuc Mouse v2 | Negative  (PPP = 0.270) | In domain |  |  |
| VEGA  (v. 1.2.8) | Micronucleus In vivo | In vivo Micronucleus activity (IRFMN) 1.0.2 | Inactive  (ADI = 0.922) | In domain | Negative | The prediction is highly reliable. Available analogues support the result. |
| OECD QSAR Toolbox 4.5 | Micronucleus In vivo | In vivo mutagenicity (Micronucleus) alerts by ISS | No alerts found | - | Not assigned | This model is a profiler that does not provide additional elements for an expert analysis. |
| VEGA  (v. 1.2.8) | Micronucleus In vitro | In vitro Micronucleus activity (IRFMN-VERMEER) 1.0.1 | Inactive  (ADI = 0.901) | In domain | Negative | The prediction is highly reliable. Available analogues support the result. |

§ The predictions may be associated with statistical value such as the PPP that is the positive prediction probability (the positive prediction probability is given as the likelihood value between 0 (non-toxic) and 1 (toxic)).

#### * Expert review: Leadscope Micronucleus In Vivo

1) Pregabalin is reported as to be negative in valid in vivo micronucleus test^2^.

2) The negative prediction given by the Leadscope In Vivo Micronuc Mouse model is driven by the correspondence of target molecule with an experimentally negative training compound

3) Low positive prediction probability is provided by the statistical model (PPP = 0.27) , meaning that, excluding the experimental data, the target molecule is predicted as negative

5) The identified model features provide a rather good coverage of the structure; they are mainly represented in experimentally negative compounds and the identified negative features provide a higher contribution to the result, resulting in an overall clear negative prediction call (PPP= 0.27). To be noted that the methylamine moiety is not covered by the features used to derive the prediction; however, no concern arise by this moiety given the negative experimental evidence of Pregabalin

6) Training set analogs were inspected and no concern arose by this analysis. Analogs are characterized by a good structural similarity with respect to the target molecule, meaning that the target molecule is well represented in the training set.

## Table S3: Summary of Pregabalin Assay Data in the NTP Integrated Chemical Environment Database

## Table S4. *In silico* predictions of endocrine activity based on the Endocrine Activity Suite provided by the Leadscope Model Applier. Negative results for both Androgen Receptor (AR) binding and Estrogen Receptor (ER) binding in rat were obtained from the ADMET predictor (not shown).

| **Model** | **Model description** | **Prediction (PPP^§^) with reliability score (RS)** |
| --- | --- | --- |
| Androgen Receptor (AR) Binding | The statistical model predicts the AR binding call which is the most conservative result of the NVS_NR_cAR, NVS_NR_hAR and NVS_NR_rAR assays^[[4]](#footnote-4)^. The results are classified as positive or negative based on hitcalls and curve fitting parameters represented in the Integrated Chemical Environment (ICE) database. The training set consists of 504 structures. | Negative (0.135) with RS =5 |
| Androgen Receptor (AR) Transactional Antagonist | The statistical model predicts the outcome of the TOX21_AR_BLA_Antagonist_ratio assay^5^. The results are classified as positive or negative based on hitcalls and curve fitting parameters represented in the Integrated Chemical Environment (ICE) database. The training set consists of 5008 structures. | Negative (0.0414) with RS =5* |
| Aromatase Inhibition | The statistical model predicts the outcome of the TOX21_Aromatase_Inhibition assay^5^. The results are classified as positive or negative based on hitcalls and curve fitting parameters represented in the Integrated Chemical Environment (ICE) database. The training set consists of 4953 structures. | Negative (0.0468) with RS =5** |
| TPO Inhibition | The statistical model predicts the Thyroperoxidase (TPO) Inhibition and more specifically the result of the Amplex Ultra Red-thyroperoxidase (AUR-TPO) assay. Data were obtained from literature. The training set consists of 850 structures. | Negative (0.306) with RS =5 |
| Estrogen Receptor (ER) Bioactivity | The statistical model predicts the result of a computational approach which integrates 18 estrogen receptor assay results^[[5]](#footnote-5)^ and determines if a chemical can act via ER agonism or ER antagonism. | Negative (0.197) with RS =5 |
| Thyroid hormone receptor (TR) binding and transactivation | The statistical model predicts the TR alpha and beta transactivation call which is the most conservative result of the TOX21_TR_LUC_GH3_Agonist and TOX21_TR_LUC_GH3_Antagonist assays^5^. The results are classified as positive or negative based on hitcalls and curve fitting parameters represented in the Integrated Chemical Environment (ICE) database. The training set consists of 6322 structures. | Negative (0.0437) with RS =5*** |

§ PPP = positive prediction probability (the positive prediction probability is given as the likelihood value between 0 (non-toxic) and 1 (toxic)).

* Experimental data available: inactive for androgen receptor antagonist from the ICE database.

** Experimental data available: inactive for aromatase inhibition from the ICE database.

*** Experimental data available: inactive for thyroid activity from the ICE database.

## Table S5: List of 22 neoplasm-related side effects with more than 25 reports involving pregabalin and disproportionality measures above 2.

| **Side Effect^1^** | **RF^2^** | **SRF^3^** | **SDRF^4^** | **PRR05^5^** | **EB05^6^** | **Alert Level^7^** |
| --- | --- | --- | --- | --- | --- | --- |
| Pituitary tumour benign | 0.02% | **81.01%** | **7.60%** | 2.63 | 2.59 | Warning |
| Metastases to bone | 0.11% | 19.72% | 2.78% | 2.06 | 2.06 | Disproportionate |
| Synovial cyst | 0.09% | 52.74% | 1.73% | 4.83 | 4.69 | Disproportionate |
| Polyp | 0.05% | 28.21% | 2.05% | 2.12 | 2.12 | Disproportionate |
| Lipoma | 0.04% | 42.86% | **6.80%** | 3.30 | 3.25 | Disproportionate |
| Melanocytic naevus | 0.04% | 11.72% | 0.69% | 2.08 | 2.09 | Disproportionate |
| Metastases to spine | 0.03% | 13.97% | 3.68% | 2.82 | 2.80 | Disproportionate |
| Malignant melanoma in situ | 0.02% | **85.11%** | 0.00% | 4.67 | 4.46 | Disproportionate |
| Haemangioma | 0.02% | 10.64% | 2.13% | 2.17 | 2.17 | Disproportionate |
| Seborrhoeic keratosis | 0.02% | 9.52% | 2.38% | 3.22 | 3.15 | Disproportionate |
| Bowen's disease | 0.02% | 1.39% | 0.00% | 3.64 | 3.52 | Disproportionate |
| Granuloma skin | 0.02% | 5.88% | 0.00% | 5.22 | 4.86 | Disproportionate |
| Renal neoplasm | 0.02% | 22.06% | **8.82%** | 2.21 | 2.19 | Disproportionate |
| Bone neoplasm | 0.01% | 56.82% | **11.36%** | 2.05 | 2.02 | Disproportionate |
| Spinal cord neoplasm | 0.01% | 55.81% | **20.93%** | 3.47 | 3.28 | Disproportionate |
| Neuroma | 0.01% | 47.62% | **7.14%** | 4.80 | 4.36 | Disproportionate |
| Monoclonal gammopathy | 0.01% | 3.03% | 0.00% | 2.67 | 2.55 | Disproportionate |
| Tonsil cancer | 0.01% | 9.38% | 3.13% | 2.28 | 2.21 | Disproportionate |
| Hypergammaglobulinaemia benign monoclonal | 0.01% | 10.71% | 3.57% | 2.49 | 2.37 | Disproportionate |
| Benign neoplasm of thyroid gland | 0.01% | 11.54% | 0.00% | 2.06 | 1.99 | Disproportionate |
| Dysplastic naevus | 0.01% | 3.85% | 0.00% | 4.13 | 3.63 | Disproportionate |
| Malignant neoplasm of unknown primary site | 0.01% | 0.00% | 0.00 | 3.78 | 3.35 | Disproportionate |

^1^ MedDRA Preferred Term

^2^ Reporting frequency

^3^ Suspicious reporting frequency (pregabalin is indicated as primary or secondary suspect); in bold those values above 70%

^4^ Single drug reporting frequency (reports mentioning pregabalin only); in bold those values above 5%

^5^ Lower 90% confidence limit for the Proportional Reporting Ratio (PRR)

^6^ Lower 90% confidence limit for the Empiric Bayes Geometric Mean (EBGM) as calculated with OpenBGM

^7^ Disproportionate means that the side effect is disproportionally reported (PRR05>2.00) for pregabalin but the values of SRF and SDRF are too low to warn a causal relationship; Warning means that beyond being disproportionally reported (PRR05>2.00), the side effect has values of SRF and SDRF that indicate a potential causal link with pregabalin.

1. Myatt, G.J., Ahlberg, E., Akahori, Y., et al., 2018. In silico toxicology protocols. Regul. Toxicol. Pharmacol. 96, 1–17. https://doi.org/10.1016/j.yrtph.2018.04.014 [↑](#footnote-ref-1)
2. Leadscope, 2022. Instem - Computational Toxicology. https://www.instem.com/solutions/insilico/computational-toxicology.php [↑](#footnote-ref-2)
3. ECHA registration dossier for 4-aminobutyric acid: <https://echa.europa.eu/it/registration-dossier/-/registered-dossier/17547/7/7/2> [Accessed in November 2021] [↑](#footnote-ref-3)
4. https://comptox.epa.gov/dashboard/assay-endpoints/ [↑](#footnote-ref-4)
5. Judson, R. S. et al. Integrated model of chemical perturbations of a biological pathway using 18 in vitro high-throughput screening assays for the estrogen receptor. Toxicol. Sci. (2015) doi:10.1093/toxsci/kfv168. [↑](#footnote-ref-5)
